# Do health workers’ preferences influence their practices? Assessment of providers’ attitude and personal use of new treatment recommendations for management of uncomplicated malaria, Tanzania

**DOI:** 10.1186/1471-2458-12-956

**Published:** 2012-11-08

**Authors:** Irene M Masanja, Angelina M Lutambi, Rashid A Khatib

**Affiliations:** 1 Ifakara Health Institute, Po Box 78373, Dar es Salaam, Tanzania; 2 Swiss Tropical and Public Health Institute, Socinstrasse 57, Basel, CH 4002, Switzerland; 3 Universität Basel, Petersplatz 1, Basel, CH, 4003, Switzerland

**Keywords:** Health workers, Attitude, Practices, New treatment guidelines, Malaria

## Abstract

**Background:**

Due to growing antimalarial drug resistance, Tanzania changed malaria treatment policies twice within a decade. First in 2001 chloroquine (CQ) was replaced by sulfadoxine-pyrimethamine (SP) for management of uncomplicated malaria and by late 2006, SP was replaced by artemether-lumefantrine (AL). We assessed health workers’ attitudes and personal practices following the first treatment policy change, at six months post-change and two years later.

**Methods:**

Two cross-sectional surveys were conducted in 2002 and 2004 among healthcare workers in three districts in South-East Tanzania using semi-structured questionnaires. Attitudes were assessed by enquiring which antimalarial was considered most suitable for the management of uncomplicated malaria for the three patient categories: i) children below 5; ii) older children and adults; and iii) pregnant women. Practice was ascertained by asking which antimalarial was used in the last malaria episode by the health worker him/herself and/or dependants. Univariate and multivariate logistic regression was used to identify factors associated with reported attitudes and practices towards the new treatment recommendations.

**Results:**

A total of 400 health workers were interviewed; 254 and 146 in the first and second surveys, respectively. SP was less preferred antimalarial in hospitals and private health facilities (p<0.01) in the first round, and the preference worsened in the second round. In the first round, clinicians did not prefer SP for children below age of 5 and pregnant women (p<0.01), but two years later, they did not prefer it for all patient scenarios. SP was the most commonly used antimalarial for management of the last malaria episode for health workers and their dependants in both rounds, in the public sector (p<0.01). Health workers in the dispensaries had the highest odds of using SP for their own treatment [adjusted OR- first round: 6.7 (95%CI: 1.9-23.4); crude OR- second round: 4.5 (1.5-13.3)].

**Conclusion:**

Following changes in malaria treatment recommendations, most health workers did not prefer the new antimalarial drug, and their preferences worsened over time. However, many of them still used the newly recommended drug for management of their own or family members’ malaria episode. This indicates that, other factors than providers’ attitude may have more influence in their personal treatment practices.

## Background

In early 2000, malaria was a leading cause of death in hospitalized patients [1] and hospital attendances in Tanzania, particularly among children under the age of five and pregnant women [2]. During that time, Tanzania recorded a range of 14–18 million clinical malaria cases yearly, with a mortality rate of 140–165 per 100,000 people and approximately 70,000 to 100,000 deaths among children under-five [1,2]. Malaria was endemic in almost all parts of the country but with varying endemicity levels [3]. Current malaria control activities in the country involve prompt diagnosis and treatment of cases, intermittent preventive treatment for pregnant women (IPTp), promoting the use of insecticide treated bed-nets (ITNs), indoor residual spraying (IRS), monitoring and managing epidemics as well as environmental management for vector control. However, the emergence and spread of insecticide and antimalarial drugs resistance may undermine the disease control strategies already in place.

The pace at which malaria parasites develop resistance to antimalarial drugs is alarming and necessitates investigating ways to prolong the lifespan of efficacious antimalarials [4,5]. The fact that drug resistance is a natural and expected process, stresses the need for cost-effective strategies to delay development of resistance, while continuing to provide effective treatment to those in need. Fear that resistance will develop to drugs in use, is justifiable and may provide a significant challenge to policymaking decisions, initiating endless cycles of drug replacement.

Towards the end of 2006, mainland Tanzania introduced Artemisinin-based combination therapy (ACTs) for routine management of uncomplicated malaria [6]. This was a second change of malaria treatment policy within a decade. The first change occurred in August 2001 when chloroquine (CQ) was replaced by sulfadoxine-pyrimethamine (SP) as the first line treatment of uncomplicated malaria [2]. The changes were necessary due to growing resistance of malaria parasites to CQ [7]. At the time of change, SP was a second line drug for treatment of malaria while quinine (QN) was reserved for severe malaria. SP was one of the few inexpensive and relatively safe antimalarial drugs that was still effective against chloroquine-resistant malaria [8]. SP was introduced on interim basis due to the fact that resistance to SP had already been recorded in some parts of the country [9-11]. Moreover, SP is known to be susceptible to resistance if used on a wide scale.

During the SP era, treatment guidelines indicated that uncomplicated malaria should be treated with SP (sulfadoxine 500 mg with pyrimethamine 25 mg) as a single dose based on age (above two months) and weight (above 5 kg) [3]. Furthermore, the policy document explained how to recognize and manage non-response to SP, contraindications, administration and adverse effects of SP. In addition, the guideline explained that SP should not be used in late pregnancy (36 weeks and above) and for lactating mothers whose children are below two months of age. Contraindications for use of SP as stated in the guideline included history of sulfadoxine hypersensitivity, premature babies and children below two months who were to receive QN. The policy also explained when and how to use the second line antimalarial drug, amodiaquine (AQ), for management of uncomplicated malaria, including indications, contraindications, adverse reactions and dosage regimen [3]. At all levels of care, severe malaria was to be managed with QN and where possible referred to a higher level of care after a pre-referral quinine shot (intra-muscular). Non responses to QN were to receive Artemisinin derivative along with QN [9].

The introduction of SP for management of uncomplicated malaria in Tanzania was received with fear and negative perceptions from both community and health care workers [12]. Fear of side effects related to sulphur content of the SP, was exacerbated by newspaper reports of suspected victims of adverse drug reactions (ADR) following use of SP. There was a chance that these fears may have affected health provider’s acceptance of the new treatment recommendations and behave inappropriately, since it is well understood that health workers’ performance can influence effectiveness of treatment policies. Their actions may be a result of their own perceptions of treatment efficacy. If a recommended treatment is perceived to be effective, it is more likely to be used as stipulated in the guideline whereas treatment recommendations that do not measure up to their expectations may be less utilized.

Contrary to expectations that health providers with more training will more likely abide by evidence based guidelines, Zurovac and colleagues reported that more qualified health workers such as clinical officers and nurses made more errors as compared to nurse aides by using non-recommended antimalarials in treating uncomplicated malaria at government health facilities in Kenya (OR 25.4; 95%CI 2.9-217.3 and OR=7.1 95%CI 1.1-44.5 respectively) [13]. In Ghana, Dodoo et al. concluded that although first line treatment recommendations may change, clinical practice can still be influenced by factors other than the decision or ability to diagnose malaria [14]. Based on a longitudinal non-intervention study to monitor adverse events, they found that age of the patient, diagnostic confirmation and suspected concurrent conditions all had significant influence in clinical practice.

Human beings tend to demand and use what is perceived to be the best available option at a given time. Clinicians and health workers would want to use the best available treatment when managing their own illnesses and their loved ones. Accurate information on this practice may shed a light on how health providers’ preferences influence their practices. The need to assess if providers’ attitude does influence their practices was particularly high following introduction of SP since; first, the change was not well received by providers and the general population, and second, the change was on an interim basis whilst evaluating other efficacious treatment options. Understanding of this behavior would assist policy making decisions for future treatment changes in Tanzania and beyond.

## Methods

### Study design

This study was completed as part of the Interdisciplinary Monitoring Project for Antimalarial Combination Treatment in Tanzania (IMPACT-TZ). Data presented here were collected in two cross sectional surveys aimed at evaluating health workers’ understanding and utilization of new treatment recommendations for use of SP as first line treatment for management of uncomplicated malaria. The evaluation was conducted in two steps: i) First survey in February 2002 (approximately 6 months post-change to SP) and ii) second survey in March 2004, two years after the first survey. In both phases, semi-structured questionnaires were used to assess health workers’ attitudes and practices related to the new recommendations.

### Site description

**Figure 1:**
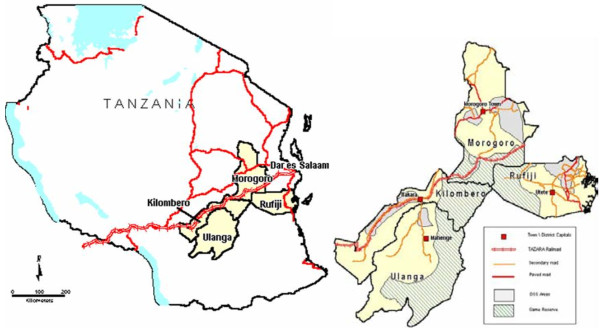
Map of Tanzania showing study sites: Morogoro Rural, Rufiji, Ulanga and Kilombero districts (Source: IMPACT- Tanzania protocol).

**Figure 2:**
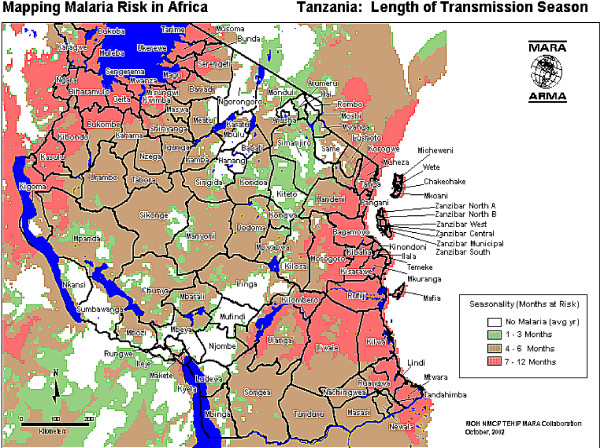
Patterns of malaria endemicity in Tanzania, 2002 (adopted from Tanzania Essential Health Interventions Project – TEHIP).

The districts involved in the evaluation were Rufiji, Morogoro Rural, Kilombero and Ulanga. For the purpose of this evaluation, Kilombero and Ulanga (K/U) were treated as a single unit because population movement between these two districts is high. Rufiji and Morogoro rural are isolated from each other and movement between them is limited by the Selous game reserve and the long distances required to by-pass the game reserve (Figure 1). The three districts are similar in terms of urban, peri-urban and rural population proportions and predicted intensity and duration of malaria transmission. Based on data modeled by the Mapping Malaria Risk in Africa (MARA) project, the three districts have a range of 7–12 months of malaria transmission season (Figure 2).

The Health and Demographic Surveillance Systems (HDSS) were in place since 1998 in Rufiji and since 1996 for Ifakara DSS (which covers the districts of Kilombero and Ulanga). In the first survey, data were collected from three districts Morogoro Rural, Rufiji and K/U. Two years later Rufiji district had started using ACT, as part of the IMPACT-TZ project evaluation and was hence excluded in the comparison between rounds.

### Sample selection

In each district, one hospital, one or two health centers and three to four dispensaries that record a high number of malaria cases were selected to participate. The health facilities were purposively chosen based on type, ownership, utilization rates and geographical location. Selection of these facilities was done by the research team in collaboration with the District Medical Officer (DMO) using district health statistics and maps.

### Eligibility criteria

Health workers from each selected facility were included in this study if they were involved in prescribing and caring for malaria patients. Interviews were conducted to eligible health workers who were present and could spare time for an interview (even after working hours), on a day of visit. In both survey rounds, the same health facilities were included, but no attempt was made to match respondents between the two surveys.

### Data collection

Information was collected through face-to-face interviews using questionnaires. Questionnaires were originally developed in English and translated into Swahili, then back translated to ensure accuracy. Trained local field workers performed interviews in Swahili and later that day, transcribed the responses into English. The field guides had two parts: first were semi-structured and open-ended questions while the second section had case scenarios. The section with semi-structured questions asked about health workers’ understanding, attitudes and practices relating to the new treatment guidelines and needed a mention of antimalarial drug from the interviewee as a response. The open-ended questions enquired about challenges and problems faced during the implementation of the new treatment policy while the case scenarios were knowledge-based questions where a patient scenario was described and required the health worker to narrate how that case would be managed. Data analyzed in this report were from the semi- structured part of the tool. Responses from the open-ended and case scenario parts will be presented elsewhere.

#### Attitudes and practices

Questions to assess attitudes asked which antimalarial drug was thought to be most suitable for management of uncomplicated malaria for: i) children below 5, ii) children above 5 years/adults and iii) pregnant women. Health workers’ practices were assessed by asking what antimalarial they or their dependants used the last time they suffered malaria.

### Data entry and analysis

Data were entered into EPI Info 2000 (CDC, Atlanta) by a project statistician and verified by the project supervisor through range and consistency checks for all variables within each dataset separately. Original questionnaires were referred to whenever inconsistency, outliers or errors were encountered. Data from the two surveys were stored in separate databases. Analysis was done using STATA 10 (Stata Corporation, Texas). The outcome variables for this paper were attitude and practice and risk factors were type of health worker, type of health facility, facility ownership and district.

### Definitions

Health workers were grouped into 5 categories; first were physicians with a minimum of a medical degree or advanced diploma in medicine, second were clinical officers (CO’s) with a diploma in medicine, third were trained nurses (TN) which included all nurses with more than two years of training i.e. registered and enrolled nurses such as nurse midwives and nurse officers. All other nurses, particularly with training of less than two years (nurse assistants and nurse auxiliaries) were grouped as “other nurses”. Other health workers e.g. laboratory assistant, who sees malaria patients only when it is necessary, were termed as “other cadres”.

Attitudes towards the national malaria treatment guidelines were assessed by comparing what health workers considered the most appropriate antimalarial for the three patient scenarios. If the response corresponded to the recommendation in the guidelines it was scored as “1” and “0” if not. Practice was assessed from the reported antimalarial used by health workers themselves or their family members in their last malaria episode. Again, if the response corresponded to the recommendation on the guideline it was scored as “1” and “0” if not. Percentages were drawn and stratified by district, type of health facility and type of health worker.

Percentages were used to describe the variables and characteristics of study participants. For each of the three scenarios, crude analyses of the associations between the various risk factors and the outcomes (attitude and practice) were performed. In the univariate analysis, cross tabulations and chi square tests were conducted to identify possible confounders. Logistic regression was used to build multivariate models to identify factors independently associated with each outcome. The group with the higher number of respondents was regarded as a reference in the multivariate analysis. Both crude and adjusted odds ratios are presented.

### Ethical approval

Ethical clearance for the IMPACT- Tanzania project was granted by the Ifakara Health Institute Review Board and Tanzanian National Institute of Medical Research (NIMR), in 2000.

## Results

### Characteristics of study participants

In the first survey, 23 health facilities were visited in the 3 districts and 254 health workers were interviewed, whereas in the second survey 16 health facilities within 2 districts were visited and 146 health workers were interviewed (Table 1). There were fewer respondents during the second survey, due to the exclusion of Rufiji district. As shown in Table 1, K/U generally had the highest percentage of participants in both phases. In the first survey, a higher percentage of participants were from non-public facilities (59%). In both rounds, hospitals had the majority of study participants compared to dispensaries and health centers (Table 1). The proportion of physicians and ‘other’ cadres was small in both surveys.

**Table 1:** Characteristics of study participants for survey rounds 1 and 2

**Characteristic variable**	**Category Total**	**Round 1**		**Round 2**	
		**n 254**	**% 100**	**n 146**	**% 100**
District	Morogoro	73	28.7	59	40.4
	Rufiji	85	33.5	-	-
	K/Ulanga	96	37.8	87	59.6
Health facility ownership	Public	105	41.3	79	54.1
	Non-public	149	58.7	67	45.9
Health facility type	Hospital	171	67.3	90	61.6
	Health center	45	17.7	22	15.1
	Dispensary	38	15	34	23.3
Cadre of health worker	Physicians	6	2.4	8	5.4
	Clinical Officers	51	20.1	26	17.8
	Trained nurses	70	27.5	55	37.7
	Other nurses	118	46.5	55	37.7
	Others	9	3.5	2	1.4

### Attitudes towards recommended treatment

Results in Table 2 shows that, in the first round, 44.1% of providers reported to prefer SP for management of uncomplicated malaria in children below five, 68% preferred SP for older children and adults and 51.6% preferred SP for pregnant women. Health workers in health centers and dispensaries were more likely to report preferring SP than in hospitals (p<0.05) and more providers from public facilities were in favor of SP than from non-public health facilities; particularly for children and pregnant women; p<0.001 (Table 2). During the second round, preference for SP treatments was very low in all categories; with only 6.2% reporting to prefer SP for management of uncomplicated malaria in children below five, 5.5% preferred SP for older children and adults, and only 4.1% preferred SP for pregnant women (Table 3). There were no statistically significant differences in reported preferences in almost all patients’ scenarios and by different providers’ characteristics (Table 3).

**Table 2:** Proportion of health workers who thought SP was appropriate treatment for uncomplicated malaria, year 2002

**Character variable**	**Category (N=254)**	**Children<5 n (%)**			**p-value**	**Older children/ adults n (%)**			**p-value**	**Pregnant women n (%)**			**p-value**
		**SP: n=112 (44.1)**	*Other drug: n=139 (54.7)	**DK/ Missing: n=3 (1.2)**		**SP: n=173 (68)**	*Other drug: n=79 (31.1)	**DK/ Missing: n=2 (0.8)**		**SP: n=131 (51.6)**	*Other drug: n=116 (45.7)	**DK/ Missing: n=7 (2.7)**	
District	Morogoro (73)	33 (45.2)	39 (53.4)	1 (1.3)	0.662	48 (65.8)	25 (34.2)	-	0.085	27 (36.9)	44 (60.3)	2 (2.7)	0.032
	Rufiji (85)	38 (44.7)	45 (52.9)	2 (2.3)		64 (75.3)	19 (22.4)	2 (2.3)		53 (62.4)	30 (35.3)	2 (2.3)	
	K/Ulanga (96)	41 (42.7)	55 (57.3)	-		61 (63.5)	35 (36.5)	-		51 (53.1)	42 (43.7)	3 (3.1)	
Health facility type	Hospitals (93)	39 (34.5)	53 (64.3)	1 (1.2)	<0.001	103 (60.2)	66 (38.6)	2 (1.2)		72 (42.1)	0.049	92 (53.8)	7 (4.1)	<0.001
	Health Centers (45)	27 (60.0)	17 (37.8)	1 (2.2)		37 (82.2)	8 (17.8)	-		31 (68.9)	14 (31.1)	-		
	Dispensaries (38)	26 (68.4)	12 (31.6)	-		33 (86.8)	5 (13.2)	-		28 (73.7)	10 (26.3)	-		
HF ownership	Public (176)	92 (52.3)	82 (46.6)	2 (1.1)	<0.001	126 (71.6)	49 (27.8)	1 (0.6)	0.189	113 (64.2)	59 (33.5)	4 (2.3)	<0.001	
	Non-public (78)	20 (25.6)	57 (73.1)	1 (1.3)		47 (60.3)	30 (38.5)	1 (1.3)		18 (23.1)	57 (73.1)	3 (3.8)		
Cadre of health worker	Physicians (6)	0	6 (100)	-	<0.001	5 (83.3)	1(16.7)	-	0.01	-	6 (100)	-	0.007	
	Clinical Officers (51)	22 (43.1)	29 (56.9)	-		38 (74.5)	13 (25.5)	-		24 (47.1)	27 (52.9)	-		
	Trained nurses (70)	25 (35.7)	45 (64.3)	-		43 (61.4)	27 (38.6)	-		30 (42.9)	38 (54.3)	2 (2.8)		
	Other nurses (118)	63 (53.4)	54 (45.8)	1 (0.8)		84 (71.2)	33 (27.9)	1 (0.9)		74 (62.7)	40 (33.9)	4 (3.4)		
	Other cadres (9)	2 (22.2)	5 (55.6)	2 (22.2)		3 (33.3)	5 (55.6)	1 (1.1)		3 (33.3)	5 (55.6)	1 (11.1)		

*Other drugs includes: Chloroquine, Amodiaquine, Artemisinins, Quinine or any combination of these P-value: Chi squared test.

**Table 3:** Health workers who thought SP was appropriate treatment for uncomplicated malaria year 2004

**Characteristic variable**	**Category (N =146)**	**Children<5 n (%)**			**p-value**	**Older children/ adults n (%)**		**p-value**	**Pregnant women n (%)**			**p-value**
		**SP; n=9 (6.2)**	*Other drug; n=135 (92.5)	**DK/ Missing; n=2 (1.3)**		**SP; n=8 (5.5)**	*Other drug; n=138 (94.5)		**SP; n=6 (4.1)**	*Other drug n=126 (86.3)	**DK/ Missing n=14 (9.6)**	
District	Morogoro (59)	1 (1.7)	58 (98.3)	0	0.08	1 (1.7)	58 (98.3)	0.09	1 (1.7)	56 (94.9)	2 (3.4)	0.04
	K/Ulanga (87)	8 (9.2)	77 (88.5)	2 (2.3)		7 (8.1)	80 (91.9)		5 (5.7)	70 (80.5)	12 (13.8)	
Health facility type	Hospitals (90)	9 (10.0)	79 (87.8)	2 (2.2)	0.11	8 (8.9)	82 (91.1)	0.07	6 (6.7)	73 (81.1)	11 (12.2)	0.18
	H/Centers (22)	0	22 (100)	0		0	22 (100)		0	21 (95.5)	1 (4.5)	
	Dispensaries (34)	0	34 (100)	0		0	34 (100)		0	32 (94.1)	2 (5.9)	
HF ownership	Public (79)	4 (5.1)	75 (94.9)	0	0.24	4 (5.1)	75 (94.9)	0.81	3 (3.8)	71 (89.9)	5 (6.3)	0.33
	Non-public (67)	5 (7.5)	60 (89.5)	2 (3.0)		4 (5.9)	63 (94.1)		3 (4.5)	55 (82.1)	9 (13.4)	
Cadre of health worker	Physicians (8)	0	8 (100)	0	0.56	0	8 (100)	0.48	0	8 (100)	0	0.41
	Cl/ Officers (26)	0	26 (100)	0		0	26 (100)		0	23 (88.5)	3 (11.5)	
	Trained nurses (55)	5 (9.1)	48 (87.3)	2 (3.6)		5 (9.1)	50 (90.9)		5 (9.1)	43 (78.2)	7 (12.7)	
	Other nurses (55)	4 (7.3)	51 (92.7)	0		3 (5.5)	52 (94.5)		1 (1.8)	50 (90.9)	4 (7.3)	
	Other cadres (2)	0	2 (100)	0		0	2 (100)		0	2 (100)	0	

*Other drugs includes: Chloroquine, Amodiaquine, Artemisinins, Quinine or a combination of these P-value: Chi squared test.

### Practices related to new recommended malaria treatment

Most health workers who had malaria episodes in the recent past had used SP for treatment in both rounds; 55% in round one and 60% in round two; indicating that, the proportion of health workers who had used SP for managing their own illnesses were higher in second survey than the first; but fewer in absolute numbers. The exception was to health workers in non-public facilities (Table 4). However, SP use for health workers dependants declined in the second round. Only providers working in dispensaries, public facilities and clinical officers recorded higher proportions of SP usage among family members (Table 4).

**Table 4:** Personal and/ or family member use of SP for management of uncomplicated malaria in 2002 and 2004

**Characteristic variable**	**Category**	**Self use N=247(%)–(2002)**		**p-value**	**Self use N=81(%) –(2004)**		**p-value**	**Own child/family N= 207(%)-(2002)**		**p-value**	**Own child/family N=84 (%) (2004)**		**p-value**
		**SP; n=135 (54.7)**	*Other drug; n=112 (45.3)		**SP; n=49 (60.5)**	*Other drug; n=32 (39.5)		**SP; n=124 (59.9)**	*Other drug; n=83 (40.1)		**SP; n=37 (44)**	*Other drug; n=47 (55)	
District	Morogoro	41 (56.2)	32 (43.8)	0.48	22 (66.7)	11 (33.3)	0.34	35 (47.9)	26 (35.6)	0.41	12 (40.0)	18 (60.0)	0.57
	Rufiji	43 (50.6)	38 (44.7)		-	-		48 (56.5)	23 (27.0)		-	-	
	K/Ulanga	51 (53.1)	42 (43.8)		27 (56.2)	21 (43.8)		41 (42.7)	34 (35.4)		25 (46.3)	29 (53.7)	
Health facility type	Hospitals	86 (50.3)	81 (47.4)	0.16	22 (50.0)	22 (50.0)	0.08	68 (39.8)	67 (39.2)	<0.001	16 (32.0)	34 (68.0)	0.01
	Health Centers	24 (53.3)	18 (40.0)		12 (66.7)	6 (33.3)		26 (57.8)	12 (26.7)		6 (50.0)	6 (50.0)	
	Dispensaries	25 (65.8)	13 (34.2)		15 (78.9)	4 (21.1)		30 (78.9)	4 (10.5)		15 (68.2)	7(31.8)	
HF ownership	Public	99 (56.3)	72 (40.9)	0.30	35 (71.4)	14 (28.6)	0.01	100 (56.8)	52(29.6)	<0.001	30 (58.8)	21 (41.2)	0.001
	Non-public	36 (46.2)	40 (51.3)		14 (43.7)	18 (56.3)		24 (30.8)	31 (39.7)		7 (21.2)	26 (78.8)	
Cadre of health worker	Physicians	3 (50.0)	3 (50.0)	0.48	2 (100)	0	0.19	2 (33.3)	2 (33.3)	0.01	0	3 (100)	0.21
	Clinical Officers	26 (51.0)	25 (49.0)		10 (83.3)	2 (16.7)		21 (41.2)	13 (25.5)		8 (66.7)	4 (33.3)	
	Trained nurses	34 (48.6)	34 (48.6)		18 (56.3)	14 (43.7)		28 (40.0)	29 (41.4)		11 (40.7)	16 (59.3)	
	Other nurses	69 (58.5)	45 (38.1)		19 (54.3)	16 (45.7)		66 (55.9)	38 (32.2)		18 (43.9)	23 (56.1)	
	Other cadres	3 (33.3)	5 (55.6)		0	0		7 (77.8)	1 (11.1)		0	1 (100)	

*Other drugs includes: Chloroquine, Amodiaquine, Artemisinins, Quinine or a combination of these.

The multivariate analysis (Table 5) shows that, compared to providers at hospitals, working at dispensaries and health centers were significant predictors of SP preference for older children and adults in the first round; adjusted OR (aOR; 95% confidence interval) = 6.3 (1.8-22.2) for dispensaries and aOR = 4.9 (1.7-14.0) for health centers (Table 5). Poor SP preference was recorded in non-public facilities with respect to management of uncomplicated malaria to pregnant women aOR= 0.2 (0.07-0.4) when compared to the public facilities. Non-public facilities had consistently lower odds of SP preferences both in a crude and adjusted analysis (Table 5). In terms of use, only providers at dispensaries had statistically significant higher odds of using SP in the last illness episode of a family member than those working in the hospitals aOR= 6.7 (1.9-23.4).

**Table 5:** Health-workers attitude and personal (or/and family) use of SP for management of uncomplicated malaria, 2002

**Characteristic variable**	**Category**	**Children<5: OR (95%CI)**		**Older children/ adults: OR (95%CI)**		**Pregnant women: OR (95%CI)**		**Self use: OR (95%CI)**		**Own child/family use: OR (95%CI)**	
	**(N=254)**	**Crude OR**	**Adjusted OR**	**Crude OR**	**Adjusted OR**	**Crude OR**	**Adjusted OR**	**Crude OR**	**Adjusted OR**	**Crude OR**	**Adjusted OR**
District	K/Ulanga (96)	Ref	Ref	Ref	Ref	Ref	Ref	Ref	Ref	Ref	Ref
	Morogoro (73)	1.1	1.5‡	1.1	0.9 ‡	0.5	0.9‡	0.9	1.1‡	0.9	0.7‡
		(0.6-2.0)	(0.6-3.6)	(0.6-2.1)	(0.3-2.2)	(0.3-0.9)	(0.3-2.1)	(0.5-1.8)	(0.5-2.4)	(0.5-1.8)	(0.3-1.6)
	Rufiji (85)	1.1	1.4‡	1.7	2.0‡	1.4	2.9‡	0.9	0.9‡	1.4	1.3‡
		(0.6-1.9)	(0.7-2.9)	(0.9- 3.3)	(0.9-4.4)	(0.8 -2.6)	(1.3-6.3)	(0.5-1.7)	(0.5- 1.9)	(0.7-2.8)	(0.6-2.7)
Health facility type	Hospitals (93)	Ref	Ref	Ref	Ref	Ref	Ref	Ref	Ref	Ref	Ref
	Health Centers (45)	2.8	2.1 Ɨ	3.1	4.9 Ɨ	3.0	2.1 Ɨ	1.2	1.2 Ɨ	1.7	1.8 Ɨ
		(1.4-5.6)	(0.9-4.7)	(1.3-6.9)	(1.7-14.0)	(1.5-6.1)	(0.9-4.9)	(0.6-2.6)	(0.5-2.6)	(0.8-3.6)	(0.6- 2.7)
	Dispensaries (38)	4.1	2.4 Ɨ	4.3	6.3 Ɨ	3.8	2.3 Ɨ	1.5	1.3 Ɨ	5.4	6.7 Ɨ
		(1.9-8.7)	(0.9-6.1)	(1.6-11.7)	(1.8-22.2)	(1.7-8.4)	(0.8-6.6)	(0.7-3.3)	(0.5-3.4)	(1.8-16.1)	(1.9-23.4)
HF ownership	Public (176)	Ref	Ref	Ref	Ref	Ref	Ref	Ref	Ref	Ref	Ref
	Non-public (78)	0.3	0.8 ¥	0.6	0.9 ¥	0.1	0.2¥	0.6	0.7 ¥	0.6	1.2¥
		(0.2-0.6)	(0.4-1.7)	(0.3-1.0)	(0.4-2.1)	(0.09-0.3)	(0.07-0.4)	(0.4-1.1)	(0.3-0.5)	(0.3-1.1)	(0.5-2.7)
Cadre of health worker	Other nurses (118)	Ref	Ref	Ref	Ref	Ref	Ref	Ref	Ref	Ref	Ref
	Physicians (6)	-	-	2.0	3.8§	-	-	0.6	0.8§	0.9	1.3§
				(0.2-17.9)	(0.4-36.4)			(0.1-3.2)	(0.1-4.5)	(0.2-5.4)	(0.2-8.2)
	Clinical Officers (51)	0.7	0.8 §	1.2	1.6§	0.5	0.7§	0.6	0.6§	1.3	1.7§
		(0.3-1.3)	(0.4-1.6)	(0.5-2.5)	(0.7-3.6)	(0.3-1.0)	(0.3-1.4)	(0.3-1.2)	(0.3-1.3)	(0.6-2.9)	(0.8-3.7)
	Trained nurses (70)	0.5	0.8§	0.6	1.2§	0.4	1.0§	0.6	0.8§	0.7	0.9 §
		(0.3-0.9)	(0.4-1.7)	(0.3-1.2)	(0.6-2.4)	(0.2-0.8)	(0.5-2.2)	(0.3-1.2)	(0.4-1.5)	(0.3- 1.2)	(0.5-1.9)
	Other cadres (9)	0.2	0.1§(0.0	0.2	0.07§	0.3	0.1§	0.5	0.4§	3.8	3.1§
		(0.04-1.2)	3-0.8)	(0.05-0.8)	(0.02-0.4)	(0.07-1.2)	(0.02-0.6)	(0.1-1.9)	(0.1-1.7)	(0.4-31.5)	(0.3-27.1)

‡Adjusted for type of health facility, health facility ownership and health worker cadre.

Ɨ Adjusted for district, health facility ownership and health worker cadre.

¥ Adjusted for district, type of health facility and health worker cadre.

§ Adjusted for district, type of health facility and health facility ownership.

In the second round (Table 6), only providers from dispensaries showed higher odds of using SP for management of their own [cOR= 5.5 (1.9-15.6)] or family member illness episodes [cOR= 4.5 (1.5-13.3)] in un-adjusted analysis, compared to providers from the hospitals. As well staff of non-public facilities showed lower odds of SP use than those working in the public facilities, in the crude analysis cOR =0.3 (0.1-0.5) for self use and 0.2 (0.06-0.5) for family member.

**Table 6:** Health workers attitude and personal use (or/and family use) of SP for management of uncomplicated malaria, 2004

**Characteristic variable**	**Category n=(146)**	**Children<5: OR (95%CI)**		**Older children/adults: OR (95%CI)**		**Pregnant women: OR (95%CI)**		**Self use: OR (95%CI)**		**Own child/family use: OR (95%CI)**	
		**Crude OR**	**Adjusted OR**	**Crude OR**	**Adjusted OR**	**Crude OR**	**Adjusted OR**	**Crude OR**	**Adjusted OR**	**Crude OR**	**Adjusted OR**
District	Morogoro (59)	Ref	Ref	Ref	Ref	Ref	Ref	Ref	Ref	Ref	Ref
	K/Ulanga (87)	5.8	3.4 ‡	5.1	2.7‡	3.5	2.4‡	1.2	0.5 ‡	1.2	2.1‡
		(0.7-48.3)	(0.3-35.2)	(0.7-42.4)	(0.2-29.9)	(0.4-31.1)	(0.2-30.7)	(0.5-2.2)	(0.1-1.8)	(0.5-3.2)	(0.5-7.8)
Health facility type	Hospitals (90)	Ref	Ref	Ref	Ref	Ref	Ref	Ref	Ref	Ref	Ref
	H/Centers (22)	-	-	-	-	-	-	1.6	0.7 Ɨ	2.1	0.8 Ɨ
								(0.6-4.4)	(0.1-3.9)	(0.6-7.6)	(0.1-4.1)
	Dispensaries (34)	-	-	-	-	-	-	5.5	1.2 Ɨ	4.5	3.0 Ɨ
								(1.9-15.6)	(0.2-8.0)	(1.5-13.3)	(0.5-17.6)
HF ownership	Public (79)	Ref	Ref	Ref	Ref	Ref	Ref	Ref	Ref	Ref	Ref
	Non-public (67)	1.5	0.5¥	1.2	0.4¥	1.2	0.5¥	0.3	0.2¥	0.2	0.2¥
		(0.4-5.8)	(0.1-2.7)	(0.3-4.9)	(0.08-2.3)	(0.2-6.0)	(0.07-3.5)	(0.1-0.5)	(0.05-1.2)	(0.06-0.5)	(0.06-1.1)
Cadre of health worker	Other nurses (55)	Ref	Ref	Ref	Ref	Ref	Ref	Ref	Ref	Ref	Ref
	Physicians (8)	-	-	-	-	-	-	1.2	2.6§(0.5-13.5)	-	-
								(0.2-5.5)			
	Clinical Officers (26)	-	-	-	-	-	-	1.9	1.4§	0.8	1.3§
								(0.7-5.4)	(0.6-3.4)	(0.3-2.3)	(0.4-4.1)
	Trained nurses (55)	1.2	0.8§	1.7	1.0§	5.4 (0.6-47.8)	3.6§ (0.3-34.9)	1.1	1.8§ (0.6-5.4)	2.5	3.1§
		(0.3-5.0)	(0.2-3.7)	(0.4-7.6)	(0.2- 5.4)			(0.4-2.8)		(0.6 -9.8)	(0.4-14.2)
	Other cadres (2)	-	-	-	-	-	-	-	-	-	-

‡Adjusted for type of health facility, health facility ownership and health worker cadre.

Ɨ Adjusted for district, health facility ownership and health worker cadre.

¥ Adjusted for district, type of health facility and health worker cadre.

§ Adjusted for district, type of health facility and health facility ownership.

## Discussion

This study provided an opportunity to assess the influence of health workers’ attitude to the usage of new malaria treatment recommendations. Overall, results showed variations in health workers attitudes and practices regarding new treatment recommendations in terms of type of health facility, ownership and type of health worker at six months post changes and two years later. There was less variation of provider’s attitudes and personal use of new recommended antimalarial between districts. Dispensaries and health centers showed higher preferences for SP than hospitals. Similarly, public facilities reported higher preference for SP than non-public ones. Most providers were not comfortable with the use of SP for children below age of 5 and for pregnant women. Personal use of SP for their malaria episodes was high in both rounds, with some exceptions in the second round.

The introduction of SP as a first line treatment of uncomplicated malaria in Tanzania was not well received [12]. It is therefore not surprising that from the first survey (baseline), preference to SP as appropriate treatment for management of uncomplicated malaria was low among health workers in the surveyed districts. Their dissatisfaction of treatment recommendation could have influenced their perceptions, attitudes and practices. This attitude may be based on their daily experiences in the clinical management of patients as became evident when we assessed providers’ preferences by type of health facility. In many areas especially rural Tanzania, hospitals are expected to be receiving referal cases. For malaria, these could mean patients who did not respond well to first line treatment at lower level of care, i.e. dispensary or health center, hence sent to hospitals for further management including laboratory assessment and in-patients’ service. This may explain the significant findings of providers from dispensaries and health workers appreciating SP use better than hospitals.

Many studies have assessed users’ perceptions of new treatments when changes occur. Several authors explored community perceptions to malaria treatment and other aspects of health services in Tanzania and elsewhere [15-17]. Likewise, most studies of health workers’ knowledge, perceptions, attitudes and understanding have been conducted in relation to health services and health problems other than malaria [18-20]. The introduction of ACTs in most African countries received considerable attention, with researchers evaluating the process of change and performance of health workers on new policies. Some of these evaluations were on artemether-lumefantrine in Kenya, Uganda, and Zambia as well as on artesunate plus amodiaquine in Ghana [14,21-23]. These evaluation assessed providers’ use of new treatment recommendations for malaria case management, with no focus on personal preferences and personal use.

The difference in providers’ preferences for SP for management of uncomplicated malaria may also be related to performance of the health facilities governing committees. A fact that public providers were more comfortable with SP than those in the non-public sector may be linked to a closer supervision of health management teams. Intrinsically health workers do assess clinical progress of their patients. Results in this survey indicate that most providers were skeptical using SP for children under 5, and pregnant women; probably because they perceived it too strong for children below age of 5 as a previous study from Tanzania reported [15].This preference worsened over time suggesting that providers were not satisfied with experiences of using SP. This finding is in contrary to what one would expect; that providers need time to appreciate, accept and comply with new treatment policies. An important lesson here is that, when there is a failing drug in the system, health care providers will, without doubt, notice it and may provide initial indication of the drug resistance in the population.

Despite poor attitudes to the new drug for first line management of uncomplicated malaria, many providers indicated that they had used SP in their last illness episode of malaria. In the first round, it was difficult to assess if SP was used before or after the change, since we did not specify the duration of illness prior the survey, but in the second round we gave a time frame; i.e. we inquired for a malaria episode in the past three months preceding the survey. Also, we did not seek additional clinical information; therefore couldn’t assess if it was correctly used. Interestingly, compared to hospitals, providers’ from the dispensaries were more likely to have used SP for their illness episode or their family members. This finding was observed in both survey rounds. One possible explanation for this observation may be related to a fact that, hospitals are a higher level of care, therefore more likely to see referal cases of malaria; i.e. non response to first line treatment and/or severe form of the disease. But also, dispensaries do not have a wider range of treatment choices and services available, hence more likely to follow treatment guidelines presented.

Also, it is more likely that, knowing this is the only available treatment option for them, dispensaries strives to have medications available in stock; hence availability of the drugs facilitated it being used by a staff or staff’s family member. This may not be a case for higher levels of care, given a wider choice of drugs available. The same may apply for public providers, with good health management team supervision, public facilities are more likely to abide by the new treatment recommendations, but this cannot be said for non-public facilities, hence significantly less use of SP for last malaria episodes was observed from non-public providers in this study. SP was available as a single dose and its price was not as high as other antimalarials available at the time of survey. During this time, other antimalarials available in private sector included amodiaquine, chloroquine (the outgoing medicine), artemisinin mono-therapy, quinine, etc. All of these products require more than a single dose to finish a course of treatment, therefore more likely to cost more than SP. A possibility of financial gain for using other treatment recommendations than SP cannot be ruled-out in the private sector.

### Study limitations

We did not account for the clustering of health facilities in the analysis. This may have affected the magnitude of the measured effect. Ideally, this clustering effect should be taken into account because there may be similarities between individuals working in the same health facility, such that, on average they are more similar to each other than to individuals in other health facilities, due to many factors such as training received at facility level and experiences acquired through everyday’s practices. However, we worked with the assumption of independence between the observations, since we were assessing individuals’ attitudes, through their preferences and personal use of treatment recommendations. These variables are more likely to be related to personal understanding and beliefs.

However, it is acknowledged that personal preferences can be influenced by many factors such as training, work experience and for the case of malaria treatment; availability of medicines and appropriate technologies to assist in clinical care of patients e.g. diagnostics for malaria confirmation, as well as presence of policy briefs and documents for referencing. These factors were not assessed and therefore limit our conclusions with regards to the role they play to shape health workers preferences and personal use of new treatment recommendations for management of uncomplicated malaria in the surveyed area.

Third, a fact that the criteria used to obtain interviewee was not random, implies that results from this evaluation cannot be generalized for all health workers in Tanzania. However, we are confident that, this study provided additional information on predictors of preferences and practices among health care providers toward SP, which complimented previous reports of poor community and provider’s perceptions towards SP when it was introduced for management of uncomplicated malaria in Tanzania; as well, it provides a clue on what happens to the health system when there is a failing drug.

Although we did not match respondents in the two surveys, the fact that we interviewed health workers from the same health facilities, increased our confidence that the differences reported in preferences and practices reflect a general picture for providers with similar experiences.

Fourth, not being able to assess clinical information when assessing practices towards recommended treatment through personal/family use, might have led to a biased estimation of SP use. It is possible that there were good reasons for not using SP to some cases that may be due to, for example, a diagnosis of severe malaria, non-response to SP or history of hypersensitivity reaction to sulphur- containing medicines.

Fifth, it is possible that some health workers reported what was considered appropriate rather than what they would actually do, or actually did, leading to courtesy bias. Furthermore, recalling what happened in terms of treating malaria in the past may have been difficult for some participants, introducing a recall bias. These biases could have affected measures of effect estimated. In this respect, we limited the recall for up to the past three months in the second survey.

Lastly, the relatively small sample for some sub-groups of explanatory variables e.g. physicians; made it difficult to detect associations between some potential risk factors and the outcomes studied in those groups.

## Conclusion

Following changes in malaria treatment guidelines, health workers in Morogoro Rural, Rufiji and K/U districts showed variations in attitude towards new recommendations. Health workers generally showed poor attitudes towards the new recommended first line treatment, but many used it in their last malaria episode or their dependants’. Clinicians did not totally accept the recommendation after the change, and this attitude worsened over time. Poor attitude to and lower use of SP for self-treatment was more apparent in hospitals and non-public health facilities. These findings indicate that other factors than provider’s attitude may play important role in providers’ practices and acceptance of new treatment recommendations. Such things as experience acquired through observation of clinical response to treatment, having a range of available treatment choices and patients’ characteristics may have more influence in clinical practices.

The need for close monitoring of implementation of new treatment policies is emphasized including assessment of training and sensitization needs for different health worker cadres and facility type, particularly early in the change process. Training should involve refresher trainings, especially in contents that seem not to be well adhered to.

## Competing interests

This work was completed as part of the cooperative agreement between the US Centers for Disease Control and Prevention and Ifakara Health Institute (IHI). Financial support came primarily from the US Agency for International Development (USAID).

Some of these data was used to fulfill requirements for a Master’s of Science (Epidemiology) degree, at the London School of Hygiene and Tropical Medicine, 2009.

## Authors’ contributions

IM- Tool translation, data collection, analysis, preparation of first draft; AML- Statistical analysis and review of draft manuscript; RAK- Supervision of data collection, review of subsequent draft manuscript. All authors read and agreed the final version of the manuscript.

## Pre-publication history

The pre-publication history for this paper can be accessed here:

http://www.biomedcentral.com/1471-2458/12/956/prepub
